# Complete Genome Sequence of *Agrobacterium tumefaciens* Ach5

**DOI:** 10.1128/genomeA.00570-15

**Published:** 2015-06-04

**Authors:** Ya-Yi Huang, Shu-Ting Cho, Wen-Sui Lo, Yi-Chieh Wang, Erh-Min Lai, Chih-Horng Kuo

**Affiliations:** a Institute of Plant and Microbial Biology, Academia Sinica, Taipei, Taiwan; b Molecular and Biological Agricultural Sciences Program, Taiwan International Graduate Program, National Chung Hsing University and Academia Sinica, Taipei, Taiwan; c Graduate Institute of Biotechnology, National Chung Hsing University, Taichung, Taiwan; d Biotechnology Center, National Chung Hsing University, Taichung, Taiwan

## Abstract

*Agrobacterium tumefaciens* is a phytopathogenic bacterium that causes crown gall disease. The strain Ach5 was isolated from yarrow (*Achillea ptarmica* L.) and is the wild-type progenitor of other derived strains widely used for plant transformation. Here, we report the complete genome sequence of this bacterium.

## GENOME ANNOUNCEMENT

*Agrobacterium tumefaciens* is a soil-dwelling bacterium that is often associated with plants. Some strains harbor a tumor-inducing (Ti) plasmid, which is required for their phytopathogenicity. During infection, the transferred DNA (T-DNA) in the Ti plasmid is integrated into the host nuclear genome. Because of this property, *A. tumefaciens* has been developed into an efficient system for plant transformation (1).

*A. tumefaciens* Ach5 was isolated from a crown gall on yarrow collected in Contra Costa County, CA, USA (2). It harbors an octopine-type Ti plasmid and could induce tumors on various woody fruit plants, as well as tobacco and tomato (3). Two notable strains have been derived from Ach5, first by Tn*904* mutagenesis to generate LBA4213 (4), and subsequently, by deletions of the T-DNA to generate LBA4404 (5). The disarmed LBA4404 is widely used for plant transformation (6). Although draft genomes are available for LBA4213 (GenBank accession numbers CP007225 to CP007228 [7]) and LBA4404 (JMKN01000001 to JMKN01000039), the genomic information of their wild-type progenitor is still lacking. To fill this gap, we report the complete genome sequence of *A. tumefaciens* Ach5 here.

The procedures for sequencing, assembly, and annotation are based on those described previously (8–16). Briefly, the Illumina MiSeq platform was used to generate 301-bp reads from one paired-end library (~391-bp insert, 8,352,732 reads) and one mate pair library (~4,150-bp insert, 9,594,498 reads). Two parallel assembly approaches were used, both using LBA4213 (7) as the reference. For the *de novo* approach, we used ALLPATHS-LG (17) to generate an initial draft. The scaffolds were oriented according to the reference and iteratively improved by PAGIT (18). For each iteration, the raw reads were mapped using BWA (19) and manually inspected using IGV (20). For the resequencing approach, the reference was used as the starting point for iterative corrections. The raw reads were mapped using BWA and polymorphisms were checked using SAMtools (21). Gaps were manually inserted at large indel sites and closed by using PAGIT. The final assembly was validated by both approaches; eight low-coverage regions were confirmed by Sanger sequencing. Gene prediction was done using RNAmmer (22), tRNAscan-SE (23), and Prodigal (24). The initial annotation was based on orthologs in *A. tumefaciens* C58 (25, 26) as identified by OrthoMCL (27). Subsequent manual curation was based on BLASTP (28) searches against the NCBI nonredundant database (29) and the KEGG database (30, 31). For the KEGG tool, we added representatives from *Agrobacterium* and *Rhizobium* (abbreviated identifiers: atu, ara, avi, ret, rec, rle, rlt, and rlg) to the default “prokaryotes” reference set.

The complete genome sequence of *A. tumefaciens* Ach5 has an overall G+C content of 58.5%. It consists of one circular chromosome (2,833,887 bp), one linear chromosome (2,095,752 bp), one mega plasmid (designated pAt; 544,752 bp), and one Ti plasmid (194,264 bp). The first version of annotation includes 15 rRNA genes, 56 tRNA genes, and 5,276 protein-coding genes.

### Nucleotide sequence accession numbers.

The complete genome sequence of *A. tumefaciens* Ach5 has been deposited at DDBJ/EMBL/GenBank under the accession numbers CP011246 to CP011249.
